# A cross-sectional study of predictors of perceptions of addiction among college students using Electronic Nicotine Delivery System users

**DOI:** 10.18332/tpc/219091

**Published:** 2026-07-16

**Authors:** Eliza Coull, Madison Fanus, Olivia LiCausi, Sharon Kingston

**Affiliations:** 1 Psychology DepartmentDickinson CollegeCarlisleUnited States

**Keywords:** Electronic Nicotine Delivery Systems (ENDS), nicotine dependence, perception of addiction, quit attempts, initiation regret

## Abstract

**Introduction:**

It is estimated that almost one quarter of young adults use electronic nicotine delivery systems (ENDS) despite health risks. Perceptions of addiction to nicotine has been linked with motivation to quit, highlighting the importance of understanding contributors to addiction perceptions among young adults.

**Methods:**

The current study used cross-sectional survey data to examine how perceptions of addiction were impacted by physical dependence, risk perception, intention to quit, number of quit attempts, and initiation regret among a sample of undergraduate students (n=99) who had used ENDS within the past 30 days. Participants were aged 18–23 years with a mean age of 19.64 years (SD=1.156). The majority (64.6%) identified as women, and 61.6% identified as White.

**Results:**

Only symptoms of nicotine dependence were significantly related to perception of addiction (β=−0.636; 95% CI: −0.829–−0.474, t=−7.415, p<0.001) indicating that experiencing more symptoms of nicotine dependence predicted stronger perceptions of nicotine addiction. The number of lifetime quit attempts moderated the relationship between symptoms of addiction and perception of addiction (β=0.129; 95% CI: 0.069–0.190, p<0.000) such that symptoms of nicotine dependence have a stronger relationship with perceptions of addiction when an individual has engaged in fewer quit attempts. The number of quit attempts did not moderate the relationship between initiation regret and perceptions of addiction or the relationship between perceptions of the risk of becoming addicted to vaping and perceptions of addiction.

**Conclusions:**

The results highlight the role of symptoms of physical dependence on perceptions of addiction among young adults attending college. Surprisingly, this relationship was stronger for individuals with fewer quit attempts. Perhaps more failed quit attempts lead young adults to adopt self-exemption beliefs and disconnect their symptoms of nicotine dependence from their perception that they are addicted to ENDS.

## Introduction

The prevalence of the use of electronic nicotine delivery systems (ENDS) among young adults is concerning, with 23.7% of individuals aged 18–25 years in the United States reporting use of ENDS in the past month^1^ and evidence that ENDS use has negative health impacts continuing to grow. ENDS use has been associated with chronic obstructive pulmonary disorder, asthma, coronary heart disease, and hypertension^2^. Studies on failed quit attempts have shown ENDS use to be highly addictive for adolescents and young adults^3,4^. Though many ENDS users have not attempted to quit, those who have attempted typically perceive themselves as addicted, and cite perceptions of addiction along with concerns about health risks as motivators to quit^5,6^. Given the association between perceptions of addiction and motivation to quit ENDS use, it is important to understand perceptions of addiction among young adult ENDS users and further explore the relationship between perceived addiction, motivation to quit, and quit attempts.

Much of the research on the connection between physical dependence on nicotine and perception of addiction has been conducted on individuals who use combustible cigarettes rather than ENDS^7-9^. Aspects of nicotine dependence, such as cravings and emotional withdrawal symptoms (i.e. mood swings, irritability)^10^ are often assessed through self-report^11^. Many individuals specifically cite signs like cravings^8,10,12^, experiencing withdrawal symptoms^12^, or developing a tolerance^3,10^, as indicators of addiction. When considering ENDS use, some users have noted that the convenience of nicotine vaping products has made it easier to quickly become dependent on the product when compared to traditional cigarettes^13^. Perceived signs of physical dependence have been found to predict individuals' beliefs that they are addicted to nicotine among both smokers and ENDS users^12^.

Given the reported ease of becoming dependent on ENDS^13^, a substantial proportion of young adults may regret starting to use ENDS. Existing literature on regret related to starting to use nicotine products, is focused on smoking cigarettes rather than ENDS usage^14,15^. A large proportion of smokers, with estimations as high as 89–90% in the United States, Canada, the United Kingdom, and Australia, and 74–93% in South Korea, Malaysia, China, and Thailand, report regretting smoking^14,15^. Regret has important implications for cessation as smoking regret is associated with intention to quit and a greater likelihood of quit attempts^14,15^.

General intentions to quit using ENDS vary among emerging adults^16-18^ with estimates ranging from 23%^19^ to 66.5%^20^ of users. Despite conflicting estimations of the proportion of ENDS users who want to quit, when asked for potential reasons to quit, responses are relatively uniform across the literature, with the most commonly cited reasons being health concerns, financial costs, and addiction^5,21-27^. When examining the relationship between intention to quit ENDS use in the future and the concept of addiction, current research often focuses on the risk of addiction to ENDS^19,23,24^, or on how that addictiveness compares to traditional cigarettes^17^, not how ENDS users perceive their own addiction (or lack thereof) to ENDS.

Studies conducted in the United States, Australia, and on English-speaking social media users, have estimated that 22.6–75.8% of ENDS users made a lifetime quit attempt, and that those who try to quit often make multiple attempts before they succeed, if they succeed at all^6,19,22,25,26^. Despite the interest in quit attempts among emerging adult ENDS users, many studies do not ask explicitly about quit attempt quantity and instead ask participants to choose from Likert scales that use descriptors like ‘lots of times’ and ‘once or twice’ to assess the number of quit attempts^6^. The few studies that have asked late adolescent ENDS users to quantify their number of quit attempts suggest that the average number of quit attempts lies somewhere between 1.9 and 2.9^25,26^. Although addiction is often reported by ENDS users as a reason for quitting^5,6,25^, research on the relationship between self-reported perceptions of addiction and actual quit attempts is scarce. Among the few studies that examine this relationship, results were mixed: one study found a possible positive relationship between self-reported addiction and quit attempts^6^, while the other found a negative relationship^27^.

Perception of addiction among emerging adults who use ENDS is an understudied phenomenon despite the possibility that it may motivate attempts to quit ENDS use^6^. It is important to understand factors that are associated with the perception of addiction to ENDS use, such as intention to quit, initiation regret, perceived risk of addiction, and symptoms of nicotine dependence. Much of the current research on these factors has been conducted on individuals who use combustible cigarettes rather than ENDS users. It is also important to understand how the number of quit attempts moderates the relationship between these factors and perceptions of addiction. The question is ‘Do failed quit attempts heighten the impact of factors related to perceptions of addiction?’.

The current study examined how perceptions of addiction were impacted by physical dependence, risk perception, intention to quit, number of quit attempts, and initiation regret among young adults who reported that they had used ENDS within the last 30 days. It was hypothesized that physical dependence, risk perception, intention to quit, number of quit attempts, and initiation regret would positively predict participants' perception of addiction. It was also hypothesized that a higher number of quit attempts would strengthen the relationships between physical dependence, risk perception, intention to quit, initiation regret, and perception of addiction.

## Methods

### Procedures

This cross-sectional study was designed to investigate ENDS use among college students. A convenience sample of students attending a small liberal arts college in the Mid-Atlantic Region of the United States was invited to participate in the study via the Psychology Department SONA system. Participants provided consent and completed the survey using Qualtrics, an online survey platform. Participants who reported that they had vaped nicotine within the past 30 days were included in the current study. All procedures were approved by the Dickinson College IRB, and participants received participant pool credit for introductory psychology classes for participation.

### Participants

The sample consists of a subset of 99 participants who reported that they had vaped nicotine within the past 30 days, taken from a sample of 574 students who completed the study on ENDS use. Demographic information about the 99 participants can be found in Table 1.

**Table 1:** Characteristics of participants in a cross-sectional study on predictors of perception of addiction among college student ENDS users, United States, 2026 (N=99)

Characteristics	% (n)
**Age** (years), mean (SD) range (18–23)	19.64 (1.16)
**Gender**	
Women	64.5 (64)
Men	33.3 (33)
Non-binary	2 (2)
**Race/ethnicity**	
White	61.6 (61)
Asian	14.1 (14)
Black	8.1 (8)
Latino(a)	7.1 (7)
Middle Eastern/North African	1 (1)
Multiracial	7.1 (7)
**Year in College**	
First-year	37.4 (37)
Sophomore	28.3 (28)
Junior	17 (17)
Senior	17 (17)
**Symptoms of nicotine dependence**, mean (SD) range (0–6)	2.07 (2.15)
**Lifetime quit attempts**, mean (SD) range (0–15)	1.61 (2.43)
**Participants reporting no quit attempts**	45.9 (45)
**I vape because I am addicted to vaping nicotine**	
Strongly agree	11.1 (11)
Agree	21.2 (21)
Neutral	12.1 (12)
Disagree	15.2 (15)
Strongly disagree	40.4 (40)
**Intention to quit**	
I don’t want to stop vaping nicotine	15.2 (15)
I think I should stop vaping nicotine but I don’t want to	16.2 (16)
I want to stop vaping nicotine but I haven’t thought about when	22.2 (22)
I really want to stop vaping nicotine but I don’t know when I will	9.1 (9)
I want to stop vaping nicotine and hope to soon	17.2 (17)
I really want to stop vaping nicotine and intend to in the next 3 months	6.1 (6)
I really want to stop vaping nicotine and intend to in the next month	14.1 (14)
**Risk of becoming addicted to vaping nicotine**	
Very unlikely	1 (1)
Unlikely	4 (4)
Likely	53.5 (53)
Very likely	41.4 (41)
**Vaping regret**	
Strongly agree	34.3 (34)
Somewhat agree	39.4 (39)
Somewhat disagree	20.2 (20)
Strongly disagree	6.1 (6)

### Measures

#### Perception of addiction

A single item, ‘Because I am addicted to vaping nicotine’, taken from the Reasons for Vaping Nicotine, a 19-item measure^28,29^, asked participants to rate reasons they vape nicotine on a five-point Likert scale ranging from 1=strongly agree to 5=strongly disagree.

#### E-cigarette-specific symptoms of nicotine dependence

A 6-item measure was adapted from the Hooked on Nicotine Checklist^30^. The current study simplified the wording of items from ‘use an e-cigarette, vape pen, or e-hookah’ to ‘vape nicotine’. The 6 items are: ‘Have you ever felt like you really needed to vape nicotine?’, ‘Do you ever have a strong urge to vape nicotine?’, ‘When you have not vaped nicotine, do you find it difficult to concentrate?’, ‘When you have not vaped nicotine, do you feel more irritable?’, ‘When you have not vaped nicotine, do you feel nervous, restless, or anxious?’ and ‘Do you typically vape nicotine within 30 min of waking in the morning?’. Respondents answer Yes=1 or No=0. Scores are summed and range from 0 to 6.

#### Perceptions of risk of addiction – nicotine vaping

The single item: ‘How likely it is that someone who vapes nicotine will become addicted to vaping?’ was rated on a Likert scale ranging from 1=Very unlikely to 4=Very likely.

#### Nicotine vaping initiation regret

A single item: ‘I wish I had never started vaping’ was rated on a Likert scale ranging from 1=Strongly agree to 4=Strongly disagree.

#### Intention to quit

Intention to quit was measured with the Motivation to Stop Scale - Nicotine Vaping (MTSS -Vaping), adapted from the Motivation to Stop Smoking Scale^31^. A single item was used that asks participants: ‘Which of the following describes you?’ with responses 1=I don’t want to stop vaping nicotine; 2=I think I should stop vaping nicotine, but I don’t want to; 3=I want to stop vaping nicotine, but I haven’t thought about when; 4=I really want to stop vaping nicotine, but I don’t know when I will; 5=I want to stop vaping nicotine and hope to soon; 6=I really want to stop vaping nicotine and intend to in the next 3 months; and 7=I really want to stop vaping nicotine and intend to in the next month. Higher scores predict a greater motivation to quit.

#### Lifetime quit attempts

A single item was used that asked: ‘How many serious attempts to quit vaping nicotine have you made in your lifetime?’.

### Statistical analysis

Descriptive statistics related to ENDS use, attitudes towards ENDS, and symptoms of nicotine dependence were analyzed using frequencies and percentages, and means and standard deviations. Multiple regression analysis, including calculation of betas and their 95% confidence intervals using SPSS version 29, was used to test the relationship between the following factors: 1) symptoms of nicotine dependence, 2) perceptions of risk of addiction, 3) nicotine vaping initiation regret, 4) intention to quit, and 5) lifetime quit attempts on the outcome variable ‘perception of addiction’. The data were examined to ensure that the assumptions of multiple linear regression were met using visual examination of scatter plots, Pearson correlations, plot analysis of residuals, and a Shapiro-Wilk test.

Hayes PROCESS for SPSS version 5.0 Model 1 was used to test three moderation models: 1) whether number of lifetime quit attempts moderated the relationship between symptoms of nicotine dependence and perception of addiction; 2) whether number of lifetime quit attempts moderated the relationship between initiation regret and perception of addiction; and 3) whether number of lifetime quit attempts moderated the relationship between perceptions of risk of addiction and perception of addiction. These analyses tested the hypotheses that the number of quit attempts would strengthen the relationship between the predictors and participants’ perceptions of addiction because a greater number of quit attempts represents failures to stop using ENDS and potentially reinforces perceptions of the intensity of nicotine dependence, the risk of dependence, and heightens initiation regret. A significance level of p<0.05 was used for the multiple regression and moderation analyses.

## Results

As a first step, descriptive statistics were run on the variables. Participants reported a mean of 2.071 (SD=2.154) symptoms of nicotine dependence, ranging from 0 to 6 symptoms, and a mean of 1.61 (SD=2.427) lifetime quit attempts, ranging from 0 to 15 quit attempts, with 45.9% of participants reporting zero lifetime quit attempts. The majority of participants (55.6%, n=55) disagreed/strongly disagreed that they vaped because they were addicted to vaping nicotine. More than one third (37.4%, n=37) reported that they intended to quit vaping sometime in the near future (defined as ‘soon’, ‘within 3 months’, ‘within the next month’) and large majorities believed that individuals are likely/very likely to become addicted if they vape nicotine (94.9%, n=94) and expressed regret that they had started vaping (73.7%, n=73) (Table 1).

The data met the assumptions of multiple linear regression. Visual examination of the scatterplots revealed linear relationships between the predictor variables and the outcome variable. A Pearson correlation matrix presented in Table 2 revealed that all correlations were below 0.80, and the variance inflation factor (VIF) for each predictor ranged from 1.129 to 1.708, indicating that no multicollinearity was present. Plot analysis of the residuals indicated that the data met the assumption of homoscedasticity and a non-significant Shapiro-Wilk=0.980 (98), p=0.132. Test of the residuals indicates that the assumption of multivariate normality was met.

**Table 2:** Correlation matrix for participants in a cross-sectional study on predictors of perception of addiction among college student ENDS users, United States, 2026 (N=99)

Predictors	1	2	3	4	5	6
**1. Perception of addiction**	-		−0.11	−0.26^b^	−0.32^c^	0.41^c^
**2. Nicotine dependence**	−0.72^c^	-				
**3. Intention to quit**	−0.11	0.15	-			
**4. Lifetime quit attempts**	−0.26^b^	0.31^c^	0.24^b^	-		
**5. Risk of addiction**	−0.32^c^	0.28^b^	0.11	−0.31	-	
**6. Initiation regret**	0.41^c^	−0.49^c^	−0.48^c^	−0.28^b^	−0.23^a^	-

a p<0.05

b p<0.01

c p<0.001

The multiple regression results indicated that the overall model explained a significant amount of variance in perception of addiction [F(5,92)=21.531, p<0.001, R^2^=0.539, R^2^_adjusted_ =0.514]. Table 3 presents the results of the multiple regression in terms of the relationship of each of the predictors to perception of addiction. Only symptoms of nicotine dependence were significantly related to perception of addiction (β=−0.636; 95% CI: −0.829–−0.474, t=−7.415, p<0.001).

**Table 3:** Regression coefficients for nicotine dependence, perceptions of risk of addiction, initiation regret, intention to quit and lifetime quit attempts for participants in a cross-sectional study on predictors of perception of addiction among college student ENDS users, United States, 2026 (N=99)

Predictors	B	SE	95% CI		β	p
			Lower	Upper		
**Constant**	5.097	0.798				**<0.001**
**Nicotine dependence**	**−0.440**	0.059	−0.558	−0.322		**<0.001**
**Perception of risk of addiction**	−0.307	0.180	−0.666	0.051	−0.128	0.092
**Initiation regret**	0.132	0.152	−0.170	0.434	0.080	0.387
**Intention to quit**	0.044	0.061	−0.078	0.166	0.059	0.477
**Lifetime quit attempts**	−0.034	0.047	−0.127	0.059	−0.056	0.469

Perception of addiction is the outcome variable

B: unstandardized coefficient; SE: standard error; β: standardized coefficient.

**Figure 1:**
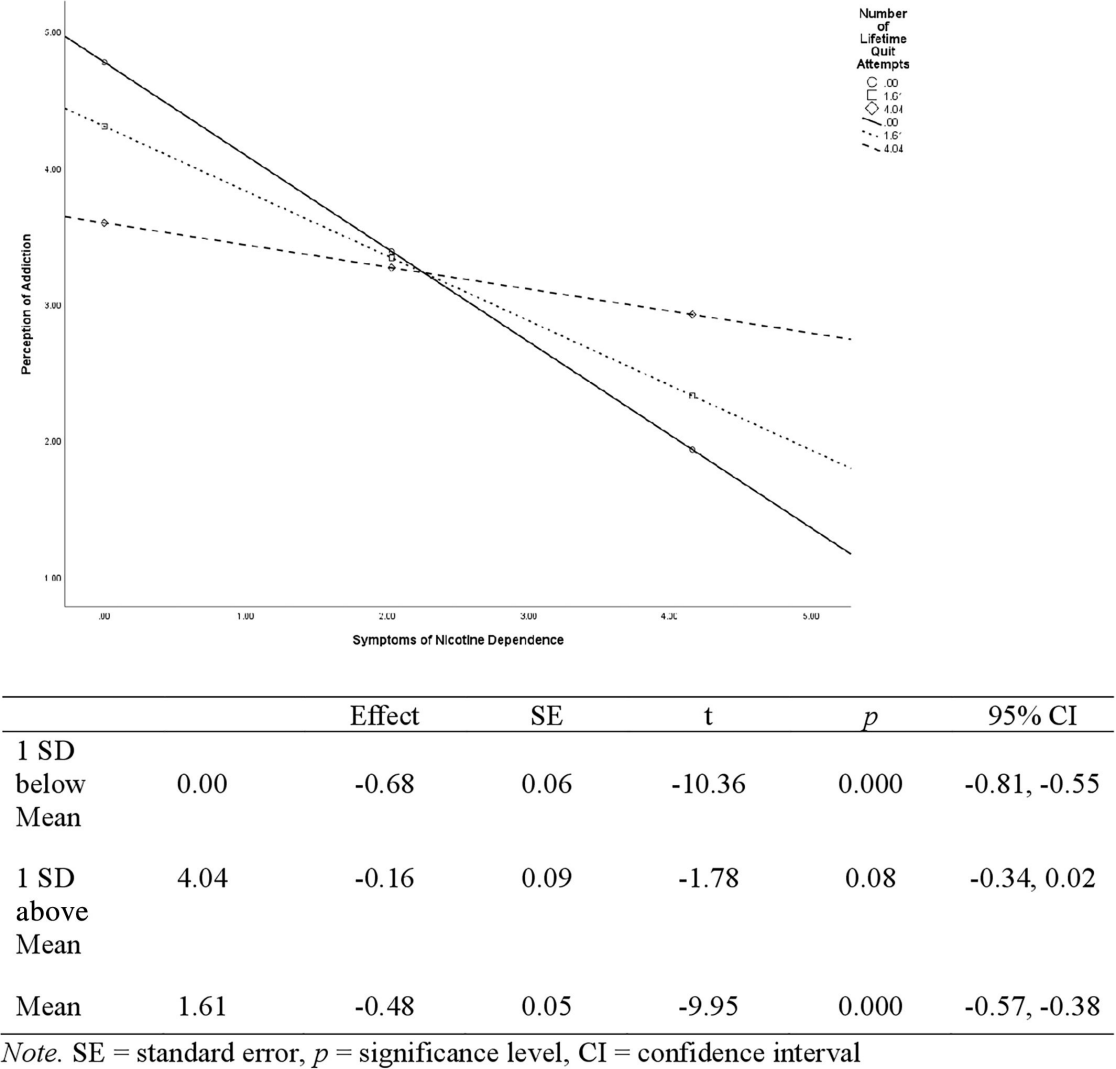
Conditional efforts of Symptoms of Nicotine Dependence at the 1 Standard Deviation below the mean Lifetime Quit Attempts, Mean Lifetime Quit Attempts and 1 Standard Deviation above the mean Lifetime Quit Attempts for participants in a cross-sectional study on predictors of preception of addiction among college student ENDS users, United States, 2026 (N=99)

In the first moderation model using ‘perception of addiction’ as the outcome variable, symptoms of nicotine dependence as the predictor, and number of lifetime quit attempts as the moderator, more symptoms of nicotine dependence significantly predicted stronger perceptions of nicotine addiction (β=−0.6834, 95% CI: −0.814–−0.552, p<0.000). A higher number of lifetime quit attempts significantly predicted stronger perceptions of addiction (β=−0.292; 95% CI: −0.442–−0.141, p<0.000). The number of lifetime quit attempts moderated the relationship between symptoms of nicotine dependence and perception of addiction (β=0.129; 95% CI: 0.069–0.190, p<0.000) such that symptoms of nicotine dependence have a stronger relationship with perceptions of addiction when an individual has engaged in fewer quit attempts (Figure 1). The conditional effects of the number of quit attempts on the relationship between symptoms of nicotine dependence and perceptions of addiction at one standard deviation below the mean, the mean, and one standard deviation above the mean of symptoms of nicotine dependence, are presented in Figure 1.

In the second moderation model with perception of addiction as the outcome variable, nicotine vaping initiation regret as the predictor, and number of lifetime quit attempts as the moderator, stronger feelings of initiation regret significantly predicted stronger perceptions of addiction (β=0.625; 95% CI: 0.263–0.987, p=0.001). The number of lifetime quit attempts did not predict perception of addiction (β=−0.053; 95% CI: −0.332–0.226, p=0.708). The number of lifetime quit attempts did not moderate the relationship between initiation regret and perceptions of addiction (β=−0.022; 95% CI: −0.161–0.118, p=0.757).

In the third moderation model using perception of addiction as the outcome variable, perceptions of the risk of becoming addicted to vaping as the predictor and number of lifetime quit attempts as the moderator, stronger perceptions of the risk of becoming addicted to nicotine significantly predicted stronger perceptions of addiction (β=−0.689; 95% CI: −1.262–0.117, p=0.018). Number of quit attempts did not significantly predict perceptions of addiction (β=−0.024, 95% CI: −0.549–0.501, p=0.928). The number of lifetime quit attempts did not moderate the relationship between perceptions of the risk of becoming addicted to vaping and perceptions of addiction (β=−0.041; 95% CI: −0.195–0.113, p<0.597). Results of the moderation models can be found in Table 4.

**Table 4:** Hayes PROCESS results of binomial regression analyses with perception of addiction as the outcome variable for participants in a cross-sectional study on predictors of perception of addiction among college student ENDS users, United States, 2026 (N=99)

Variable	β	SE	t	p	95% CI
**Relationship between symptoms of nicotine dependence and perception of addiction with number of lifetime quit attempts as moderator**					
Symptoms of nicotine dependence	**−0.68**	0.06	−10.36	**0.000**	−0.81 – −0.55
Number of lifetime quit attempts	**−0.29**	0.08	−3.85	**0.000**	−0.44 – −0.14
Nicotine dependence×Quit attempts	**0.13**	0.03	4.24	**0.000**	0.07 – 0.19
**Relationship between initiation regret and perception of addiction with number of lifetime quit attempts as moderator**					
Initiation regret	**0.62**	0.18	3.43	**0.001**	0.26 – 0.99
Number of lifetime quit attempts	−0.05	0.14	−0.38	0.71	−0.33 – 0.23
Initiation regret×Quit attempts	−0.02	0.07	−0.31	0.76	−0.16 – 0.12
**Relationship between symptoms of perception of risk of becoming addicted and perception of addiction with number of lifetime quit attempts as moderator**					
Perceptions of risk of addiction	−0.69	0.29	−2.39	0.02	−1.26 – −0.12
Number of lifetime quit attempts	−0.02	0.26	−0.09	0.93	−0.55 – 0.50
Risk of addiction×Quit attempts	−0.04	0.08	−0.53	0.60	−0.19 – 0.11

SE: standard error; β: standardized coefficient.

## Discussion

The results indicated that while the majority of the sample of current ENDS users perceived that vaping nicotine posed a risk of addiction and regretted initiating ENDS use, less than half of the participants believed that they themselves were addicted to nicotine or intended to quit using ENDS in the near future. In the multivariate analyses, only symptoms of nicotine dependence predicted perception of addiction. A lower score on the perception of addiction item indicates stronger agreement that one is addicted to nicotine, and the results of the multivariate analysis indicate that individuals with more symptoms of nicotine dependence were more likely to report that they agreed that they used ENDS because they were addicted to nicotine. Contrary to the hypotheses, the number of lifetime quit attempts did not strengthen the relationships between nicotine dependence, initiation regret, or perceptions of risk of addiction and participants’ perception that they were addicted to vaping nicotine. The number of lifetime quit attempts did moderate the relationship between symptoms of nicotine dependence and perceptions of addiction, but not in the expected direction. Symptoms of nicotine dependence were more strongly related to perceptions of addiction among individuals with fewer lifetime quit attempts.

Consistent with previous research, physical dependence was a significant predictor of perception of addiction. This finding is consistent with literature on the role of physical dependence among ENDS users, where higher rates of nicotine dependence are associated with a higher likelihood of perceived addiction^11^. For instance, users often report symptoms of physical dependence when asked whether or not they are addicted to nicotine (i.e. cravings, withdrawal symptoms, heightened tolerance, and/or increased usage)^3,8-10,12^. ENDS users are also at increased risk of developing a dependence on nicotine, as ENDS use can produce rapid and high levels of nicotine when compared to traditional cigarette smoking^32^. In the current study, initiation regret and perception of risk of addiction did not predict participants’ perception that they themselves were addicted in the multivariate analysis. This finding aligns with research that shows that current, short-term benefits of ENDS use tend to outweigh the negative effects of use. Often, ENDS users believe that the negative consequences of vaping do not apply to them (i.e. because vaping is safer than smoking, their age, their duration of usage, etc), indicating self-exemption beliefs that impact usage and reduce desire for cessation. Users frequently do not perceive their use as problematic or believe that the benefits outweigh the risks – until they personally experience symptoms of physical dependence. At that point, they often begin to view their addiction as a problem, which can motivate a desire to quit^4,14^.

Although there are a limited number of studies examining the relationship between quit attempts and self-perception of addiction, and the existing results are mixed, the finding that a higher number of lifetime quit attempts were not related to stronger perceptions of addiction in the multivariate model is consistent with a longitudinal study examining adults from Australia, Canada, the United States, and England that found that perceiving oneself as addicted to ENDS was associated with fewer quit attempts^27^. The results of the current study are inconsistent with the findings of a cross-sectional study that examined Australian youth between the ages of 14 and 25 years, which found a positive relationship between self-reported ENDS addiction and quit attempts^6^. The Australian study measured the number of quit attempts by asking participants if they had attempted to quit vaping and providing a three-point scale ranging from ‘yes, lots of times’, ‘yes, once or twice’ to ‘no’ as opposed to the current study, which asked participants to provide the number of quit attempts they had made in their lifetime. The difference in measurement approaches may explain the lack of consistency between the results.

Further complicating our understanding of the relationship between the number of quit attempts and perceptions of addiction, the number of quit attempts did not moderate the relationship between initiation regret or perceptions of risk of addiction and participants’ perception that they were addicted to vaping nicotine. It was hypothesized that more failed attempts to quit would heighten the relationships between initiation regret and perceptions of the risk of addiction and perception of addiction. The number of quit attempts did moderate the relationship between perceptions of physical dependence on nicotine and perception of addiction, but not in the predicted direction. It was hypothesized that the relationship between perceptions of physical dependence on nicotine and perceptions of addiction would be stronger among individuals who reported more quit attempts; instead, the relationship was stronger for individuals with fewer quit attempts. No other study examining the number of quit attempts as a moderator of predictors of perceptions of addiction among late adolescent or young adult ENDS users was found in the peer-reviewed literature at the time of the current study. Prior research does suggest that adolescents may not have a clear definition of the concept of addiction^9^. Perhaps late adolescents and young adults who have experienced more failed quit attempts begin to adopt self-exempting beliefs that lead them to disconnect their experience of physical dependence from their perception of their own addiction.

While the current study focused on how lifetime quit attempts impacted the addiction perceptions of young adult ENDS users, future research should focus on how ENDS users are impacted by each individual quit attempt. As previously mentioned, ENDS users who attempt to quit typically do so more than once^25,26^, and while some studies have examined how quit intentions change over time^19,23^, there are no studies to our knowledge examining whether attitudes and perceptions change after each quit attempt. This future research direction is especially important when considering cessation interventions and self-exemption beliefs among nicotine users, as previous research suggests that self-exemption beliefs surrounding the health risks of nicotine are common^33^. Without future research examining ENDS users’ attitudes after each quit attempt, it is difficult to create cessation support programs and understand whether numerous unsuccessful quit attempts strengthen self-exemption beliefs and discourage intentions to try quitting again or whether they increase risk perceptions and strengthen intent to quit.

Information pertaining to the potential harm associated with ENDS use (e.g. lung damage) is commonly included in interventions designed to discourage ENDS use among adolescents and emerging adults, and has been shown to be somewhat effective^34,35^. However, there are numerous instances where education regarding the risks of ENDS use does not decrease ENDS use among young adults^35^. The current results highlight the importance of educating individuals about the signs of nicotine dependence, since recognizing those symptoms increases an individual’s perception that they are addicted to nicotine, and recognition of addiction can act as a key motivator for ending nicotine use^5^.

### Limitations

The current study had several limitations. First, it was a cross-sectional study. The lack of randomization and longitudinal data makes it impossible to infer causality or the directionality of the relationships between the variables. All of the participants were late adolescents or early adults enrolled in college. The results may not generalize to other age ranges. Individuals in this age range may have distinct self-exempting beliefs, specifically, the belief that the individual will be able to quit in the future and avoid negative health impacts. In addition to age, the sample was predominantly White. Research on more racially and ethnically diverse samples is needed. Furthermore, while the study was adequately powered with 20 participants per predictor for the multiple regression, a larger sample size might have been able to detect small effects.

In terms of measurement, all measures were self-reported, making them vulnerable to social desirability and recall biases. In addition, the measure of the number of quit attempts used in the current study may not fully capture attempts to permanently stop using ENDS. Specifically, definitions of a ‘quit attempt’ may vary among individuals. For example, qualitative interviews with a subsample of current vapers in the sample revealed that some collegiate athletes reported that they did not use ENDS during their sports season. It is unclear if they considered those periods as quit attempts. Finally, the criteria for distinguishing current ENDS users were ENDS use within the past 30 days. This is a commonly used measure of current use in research on substance use; however, due to the age of the sample, that definition might have included people who had recently initiated use and were experimental or infrequent social users. This definition of current use may have resulted in a larger proportion of individuals who are not experiencing physical dependence in an older population.

## Conclusions

The results highlight the role of symptoms of physical dependence on perceptions of addiction among young adults attending college. Young adults who reported more symptoms of physical dependence were more likely to perceive that they were addicted to ENDS use. Surprisingly, this relationship was stronger for individuals with fewer quit attempts. Future research should incorporate longitudinal designs to better understand the factors related to young adults perceiving that they are addicted to ENDS use and focus on how young adults interpret the meaning of failed quit attempts in order to inform interventions that support young adults who use ENDS to continue their cessation efforts.
